# Protomix: a Python package for ^1^H-NMR metabolomics data preprocessing

**DOI:** 10.1093/bioadv/vbae192

**Published:** 2024-11-27

**Authors:** Mohammed Zniber, Youssef Fatihi, Tan-Phat Huynh

**Affiliations:** Laboratory of Molecular Science and Engineering, Åbo Akademi University, Henrikinkatu 2, Turku 20500, Finland; Department of Computer Science, Ibn Tofail University, Kenitra 14000, Morocco; Laboratory of Molecular Science and Engineering, Åbo Akademi University, Henrikinkatu 2, Turku 20500, Finland

## Abstract

**Motivation:**

NMR-based metabolomics is a field driven by technological advancements, necessitating the use of advanced preprocessing tools. Despite this need, there is a remarkable scarcity of comprehensive and user-friendly preprocessing tools in Python. To bridge this gap, we have developed Protomix—a Python package designed for metabolomics research. Protomix offers a set of automated, efficient, and user-friendly signal-preprocessing steps, tailored to streamline and enhance the preprocessing phase in metabolomics studies.

**Results:**

This package presents a comprehensive preprocessing pipeline compatible with various data analysis tools. It encompasses a suite of functionalities for data extraction, preprocessing, and interactive visualization. Additionally, it includes a tutorial in the form of a Python Jupyter notebook, specifically designed for the analysis of 1D ^1^H-NMR metabolomics data related to prostate cancer and benign prostatic hyperplasia.

**Availability and implementation:**

Protomix can be accessed at https://github.com/mzniber/protomix and https://protomix.readthedocs.io/en/latest/index.html.

## 1 Introduction

Metabolomics is an emerging field within systems biology and represents the final step in the ‘omics’ cascade (Euceda *et al.* 2015, Nagana Gowda 2019). As a technology-driven discipline, metabolomics utilizes advancements in analytical chemistry and computational techniques to improve data collection, analysis, and interpretation (Emwas *et al.* 2019). Key platforms in metabolomics, such as nuclear magnetic resonance (NMR) and mass spectroscopy (MS) (Nagana Gowda and Raftery 2023), require sophisticated data preprocessing tools to address experimental variations. It is important to note that incomplete or improper data preprocessing can hinder subsequent data analysis, resulting in inaccurate findings (Liland 2011). Although commercial tools are commonly employed for ^1^H-NMR data preprocessing, they often lack advanced editing features, requiring manual adjustments that can affect data quality. Open-source alternatives provide greater flexibility and customizability, yet they face challenges in terms of irregular updates, compatibility, and data transfer issues, and noninteractive visualization, which can obstruct data preprocessing. There are several preprocessing tools such as NMRSpec (Liu *et al.* 2017), Speaq (Beirnaert *et al.* 2018), PepsNMR (Martin *et al.* 2018), MVAPACK (Worley and Powers 2014), Metabolab (Ludwig and Günther 2011), and Workflow4Metabolomics (Giacomoni *et al.* 2015). However, despite the widespread use of Python in data analysis, the literature about NMR preprocessing packages in Python is scarce. The existing packages are often outdated, not specifically designed for metabolomics studies, or lack interactive visualization capabilities (Helmus and Jaroniec 2013, Fitzpatrick *et al.* 2014). This highlights the need for more accessible and well-documented tools to further metabolomics research.

In this article, we report the development of a comprehensive Python library named Protomix (‘Proto’ from proton nuclear magnetic resonance and ‘omix’ from metabolomics), tailored to streamline and improve the data preprocessing phase in ^1^H-NMR metabolomic studies. Protomix offers a range of preprocessing steps, interactive charts, and user-friendly features, making it accessible even to those with limited programming experience.

## 2 Methods

**Figure 1.**
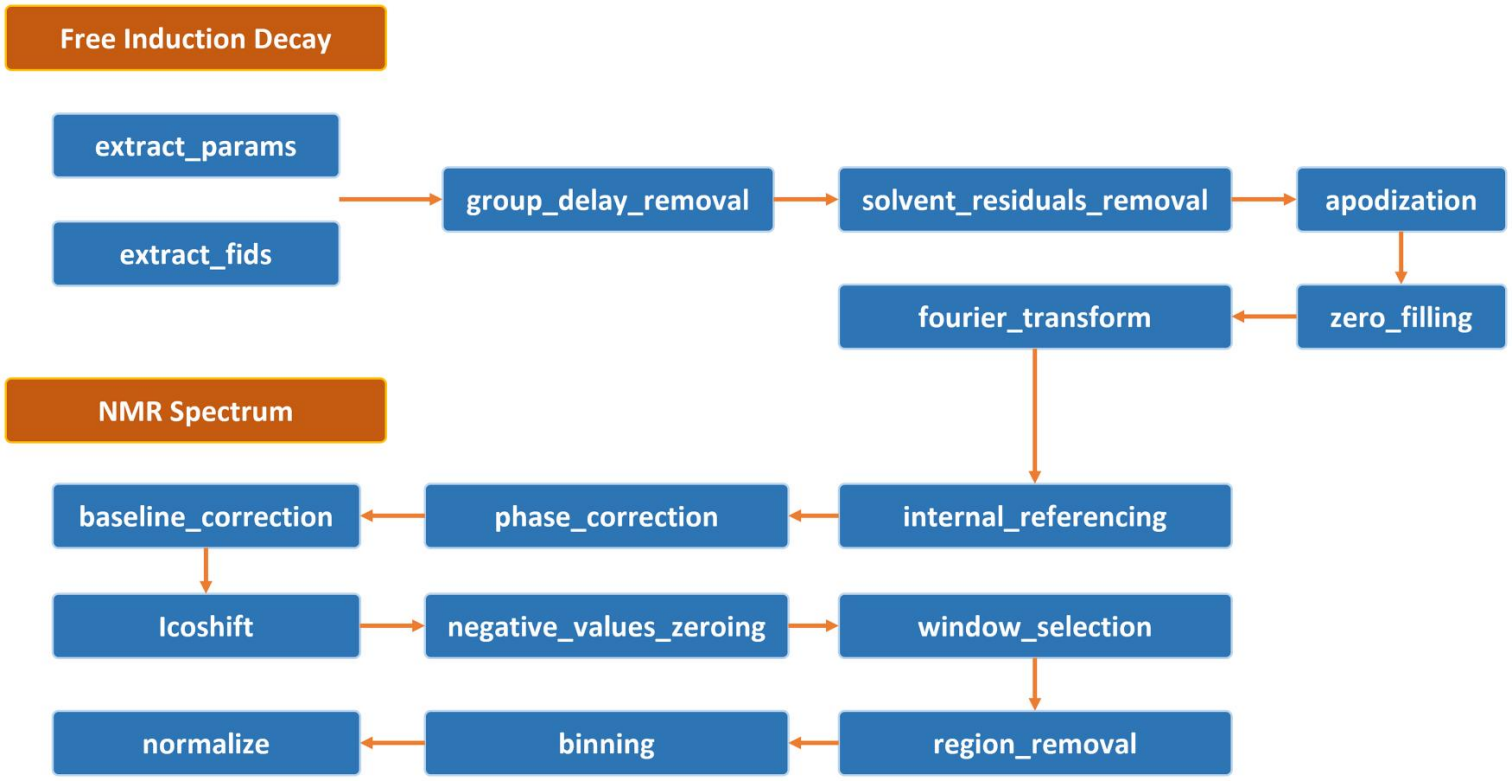
Step-by-step workflow for NMR metabolomics data preprocessing using Protomix.

Protomix is an open-source package accessible via the Python Package Index (PyPI), providing a comprehensive set of preprocessing steps facilitating the conversion of free induction decay (FID) into preprocessed spectra. Throughout the preprocessing phase, users have the advantage of interactive data visualization features provided by Plotly, a Python package for data visualization, enabling real-time monitoring of ongoing processes. A detailed depiction of this preprocessing package is illustrated in Fig. 1.

### 2.1 Raw signal

Each FID (as depicted in Supplementary Fig. S1) denotes a complex decaying signal represented as *S* = *Sx* + i*Sy*, comprising *n* data points, where *Sx* and *Sy* denote the real and imaginary parts of the signal, respectively. Each individual signal can be written as a function of its offset (*ν*), amplitude (*S*_0_), and relaxation time (*T*) over the time span (*t*) (Akitt and Mann 2017), formulated as:

$$S=S_{0}\exp^{i2\pi \nu t}\exp^{\left(-\frac{t}{T}\right)}$$

To facilitate the extraction of such signals, two primary functions are employed: the first function (‘extract_params’) is designed to extract parameters from ‘acqus’ files found within a specified directory. It scans through the directory to find all ‘acqus’ files and reads the parameters contained in these files. These parameters are then compiled into a pandas DataFrame, indexed by the samples’ names, which are derived from the directory names containing the ‘acqus’ files. While the second function (‘extract_fids’) is utilized to extract the FID data from ‘fid’ files found in the specified directory. It works in conjunction with the previous function, using the Acqus DataFrame as one of its inputs.

### 2.2 Group delay removal

The ‘group delay’ or ‘death time’ notably observed in Bruker spectra is considered as a bug due to its significant effect on the spectral quality, making it noisy. This artifact introduces a first-order phase error in the spectrum, originating from the digital decimation and filtering processes involved in high-frequency oversampling (Cobas 2008). The removal of group delay can be achieved by directly eliminating the data points corresponding to the group delay from each FID (Supplementary Fig. S2). This function (‘group_delay_removal’) facilitates this process efficiently; it extracts the group delay parameter from the ‘acqus’ DataFrame and subsequently removes the corresponding data points from the FID of each spectrum without the necessity of complex transformations.

### 2.3 Solvent residuals removal

Water in biological samples chemical shift changes according to experimental conditions, exhibiting a broad resonance peak (Supplementary Fig. S3). Several solvent suppression techniques exist, aiming to reduce the magnitude of the water resonance, thereby unveiling the NMR signal of the compounds of interest. However, a small number of residuals still remain, masking other compounds and hindering phase and baseline corrections. The given function (‘solvent_residuals_removal’) removes solvent residuals by using discrete penalized least squares with second-order differences based on Whitaker’s smoother (Whittaker 1922, Eilers 2003).

### 2.4 Apodization

Apodization is a mathematical process applied to NMR data before Fourier transformation, aimed at enhancing both the resolution and signal-to-noise ratio of the resulting spectra. This is achieved by multiplying the FID with a decaying mathematical function, which can help in reducing noise and enhancing the spectral quality. It is important to note that the intensity of the FID signal decreases as time progresses, in contrast to noise which exhibits constant fluctuations. Consequently, the signal-to-noise ratio is higher at the beginning of the FID and decreases gradually with time. This procedure represents a trade-off between resolution and sensitivity, and the choice of the function can significantly influence the quality of the resulting spectra (Supplementary Fig. S4). An apodization function provided in Protomix, applies an apodization function (‘apodization’) (either Gaussian or exponential) to the rows of a pandas DataFrame that contains FIDs (Ebel *et al.* 2006, Martin *et al.* 2018).

### 2.5 Zero filling

This function (‘zero_filling’) improves data quality by increasing the number of data points to the end of the FID before the Fourier transform (FT). This process only enhances the spectra peaks and does not alter the data (Ebel *et al.* 2006).

### 2.6 Fourier transform

The FID acquired in the time domain, undergoes a transformation to the frequency domain to form an NMR spectrum through the application of the FT. This process extracts peaks characterized by distinct heights, positions, and widths (Supplementary Fig. S5), which are determined by the respective amplitude and relaxation time of the signal. Protomix provides an FT function, utilizing the NumPy library to facilitate this transformation. This function (‘fourier_transform’) employs the discrete FT to analyze uniformly spaced samples through the fast Fourier transform (FFT) algorithm. Subsequent to this transformation, the frequency values are translated to the chemical shift range (Günther 2013).

### 2.7 Internal referencing

Aligning samples with a reference compound at 0 ppm is standard practice (Supplementary Fig. S6). This step not only helps in the identification and quantification of metabolites but also enables the comparison of NMR spectra. Two common references often employed for this purpose are 3-(trimethylsilyl) propionic-2,2,3,3-d4 acid sodium salt (TSP) and 2,2-dimethyl-2-silapentane-5-sulfonate-d4 sodium salt (DSS). The given function (‘internal_referencing’) in this step calibrates the NMR spectra by locating the reference compound peak within a ppm range defined by two parameters: ppm_min and ppm_max, which establish the ppm search range with respective default values of -0.2 and 0.2.

### 2.8 Phase correction

Theoretically, it is presumed that at the start of the FID at time zero, the *Sx* component reaches its peak, while the *Sy* component of the signal is null. However, practically, this scenario may not always hold true, leading to what is termed as a phase shift or phase error in the signal. Consequently, the real part of the spectrum fails to exhibit a pure absorption lineshape, which is necessary to achieve optimal resolution (Supplementary Fig. S7) (Keeler James 2002).

$$S(t)=S_{0}\exp^{i2\pi \nu t}\exp^{\left(-\frac{t}{T}\right)}\exp^{i\Phi_{\mathrm{error}}}$$

The phase error can be removed by multiplying the shifted signal by $\exp^{i\Phi_{\mathrm{correction}}}$ where $\Phi_{\mathrm{correction}}=\;\Phi_{\mathrm{error}}$.

$$S_{\mathrm{corrected}}\left(t\right)=S_{0}\exp^{i2\pi \nu t}\exp^{\left(-\frac{t}{T}\right)}\exp^{i\Phi_{\mathrm{error}}}\exp^{-i\Phi_{\mathrm{correction}}}$$

The provided function (‘phase_correction’) is designed to find the optimal correction angle by rotating each spectrum in the DataFrame and optimizing the root mean square (RMS) of the positive peaks in the spectrum (Martin *et al.* 2018).

### 2.9 Region removal

Spectral regions containing interfering signals are removed from the spectra to prevent hindrance in subsequent data analysis. In the case of biological samples, the water resonance peak is considered irrelevant, often inducing unwanted distortions in the water resonance area during phase and baseline corrections. To address this, a region removal function (‘region_removal’) is employed to eliminate these regions. For urine samples, it is advised to remove peaks linked to urea, maleic acid, and water, which span from 4.5 to 6.1 ppm (Supplementary Fig. S8), while for serum samples, it is practical to exclusively remove the water resonance region between 4.5 and 5.1 ppm.

### 2.10 Baseline correction

This step focuses on correcting baseline distortions that are due to incomplete relaxation or imperfections in the pulse sequences, magnetic field inhomogeneities, experimental conditions, solvent effect, phase errors, and the presence of macromolecules (Supplementary Fig. S9) (Xi and Rocke 2008, Euceda *et al.* 2015). In this function (‘baseline_correction’), we’ve incorporated three methods adapted from the pyBaselines package: asymmetric least squares (ALS), adaptive reweighted penalized least squares (ARPLS), and automatic iterative reweighted penalized least squares (AIRPLS). While these are the included methods, the pyBaselines package offers a variety of strategies that users can explore and utilize (Erb 2022).

### 2.11 Peak alignment

Inherent characteristics of biological samples as well as variations in experimental conditions including pH, temperature, or concentration of biological components often result in peak shifts across or misalignments among identical features across different spectra. Several algorithms exist for peak alignment (Vu and Laukens 2013), but in this package, we have specifically focused on the Icoshift algorithm (Savorani *et al.* 2010). This algorithm operates on the principle of FFT cross-correlation, shifting spectral intervals to achieve simultaneous alignment of all spectra (Supplementary Fig. S10). A MATLAB implementation exists, but we did not convert it ourselves. Instead, we utilized a Python implementation of this algorithm (‘Icoshift’) (Krossa 2022).

### 2.12 Negative values zeroing

Even following baseline and phase corrections, spectra might still contain negative values. This function (‘negative_values_zeroing’) effectively nullifies all such negative values to zero, as they cannot be interpreted.

### 2.13 Window selection

This function (‘window_selection’) is employed to extract a particular spectral window, defined by chemical shift boundaries (default parameters: min: 0.2 ppm and max: 10 ppm). This procedure diminishes the number of data points in the spectrum, allowing for a focused analysis on information-rich spectral regions.

### 2.14 Binning

Binning is a simple yet effective method for reducing the high dimensionality of NMR spectra and remove residual peak shifts prior to multivariate statistical analysis. Binning splits the data into small regions, often including one or more NMR peaks. The provided code defines a function (‘binning’) that performs equidistant binning on NMR spectra by integrating the area under the curve for each bin (Izquierdo-García *et al.* 2011).

### 2.15 Normalization

Normalization is a popular data processing technique that ensures comparability across all samples. In the case of biological fluids, serum and plasma do not necessitate correction for dilution effects owing to their strict physiological control. On the contrary, urine, which is significantly affected by fluid intake along with various physiological and physio-pathological factors, exhibits notable variations in concentration of metabolites’. The concentrations of endogenous metabolites in urine can fluctuate by several orders of magnitude, even within the same individual. Thus, it is important to make adequate corrections for these intensity/concentration variations (Dieterle *et al.* 2006, Emwas *et al.* 2018). This package offers a normalization function (‘normalize’) based on probability quotient normalization (PQN), total area, and standard normal variate, aiming to normalize spectra by correcting dilution differences while respecting data variability.

## 3 Results

**Figure 2.**
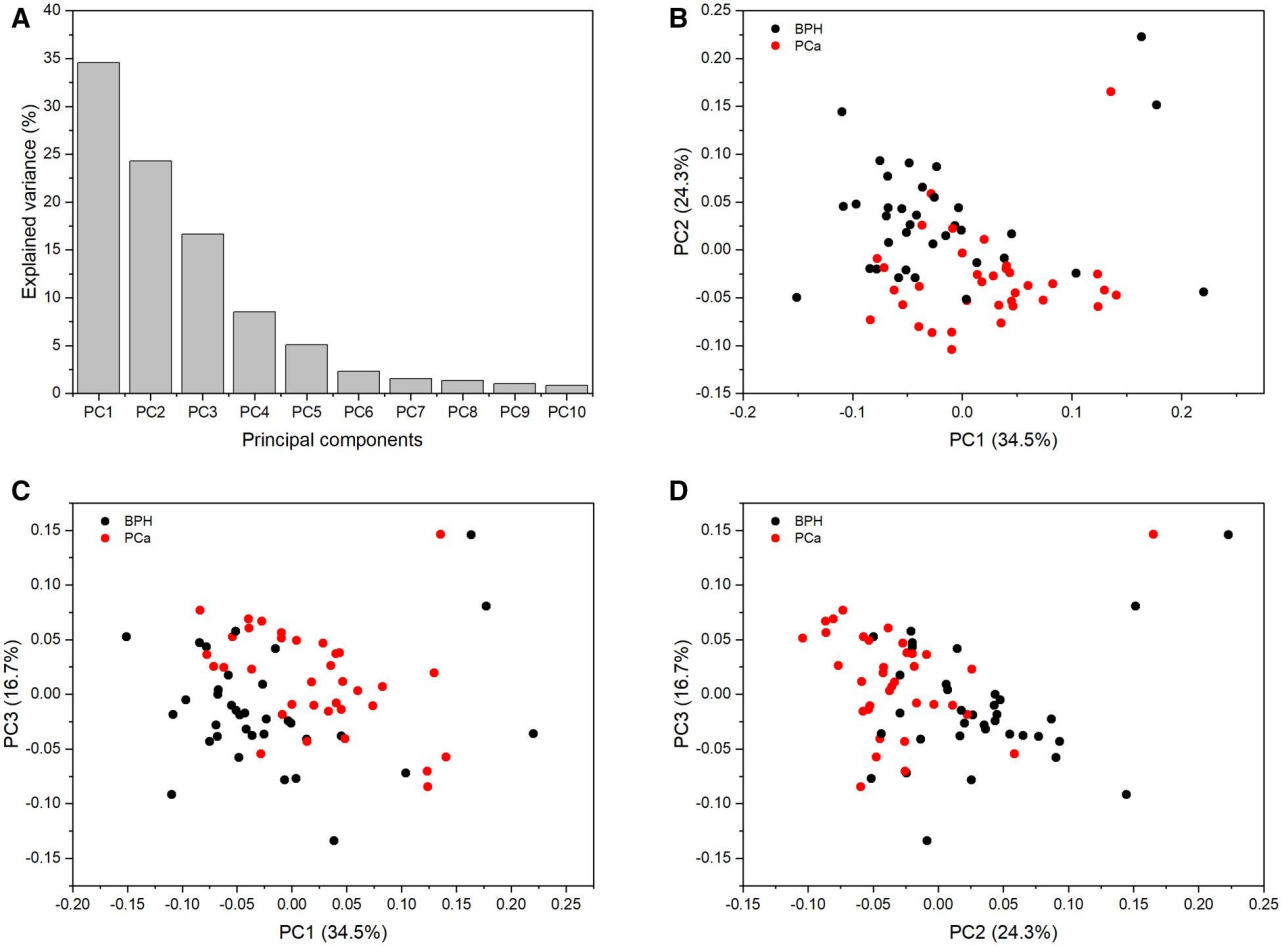
Variance explained by each principal component (A). Score plots of PC1 versus PC2 (B), PC1 versus PC3 (C), and PC2 versus PC4 (D).

In our study, we employed Protomix for the analysis of one of our previous ^1^H-NMR datasets that included 70 urine samples from patients diagnosed with prostate cancer and benign prostatic hyperplasia (Zniber *et al.* 2024a,b). Further information about this data and how it was acquired can be found in Supplementary Material. The preprocessing of this dataset (more details in the tutorial) was efficiently completed in under two minutes, utilizing an 8-core laptop. Next, the above-mentioned pre-processed spectra were analyzed using principal component analysis (PCA). Figure 2 illustrates the contribution of each principal component towards capturing the total variance in the data. It can be seen that the first 10 components collectively account for a substantial portion, approximately 92.38%, of the total variance (Fig. 2A). The diminishing explained variance after the two initial components indicates that the majority of the variability is captured by the first few components, suggesting that a reduced set of dimensions might be enough to represent the main features of the data. Moreover, the score plots illustrated in Fig. 2B–D provide a visual representation of the samples in the reduced space defined by the principal components. Also, the loading plots for PC1, PC2, PC3, and PC4 offer detailed insights into the contribution of each bucket to these components, further enriching our understanding of the data structure (Supplementary Fig. S11). Noteworthy patterns emerge, such as PC1, PC2, PC3, and PC4, which predominantly encapsulate a significant portion of the biological variability among the samples. These components collectively capture approximately 68.25% of the total variability present in the dataset. This emphasizes the relevance of these specific components in capturing essential biological features within the dataset.

In conclusion, the development of Protomix represents a significant advancement in the preprocessing of ^1^H-NMR metabolomics data in Python. This package not only simplifies the preprocessing phase but also improves it through interactive data visualization, offering an accessible tool for researchers with varying levels of programming expertise. This article addresses the need for more user-friendly and comprehensive open-source preprocessing tools, thereby broadening the accessibility and application of advanced computational methods in metabolomics. The ongoing development and refinement of Protomix will further strengthen its role as a vital tool in the fields of metabolomics.

## Supplementary Material

vbae192_Supplementary_Data

## Data Availability

Project name: Protomix. Project home page: https://github.com/mzniber/Protomix Platform: Binaries are available for download on Windows and MacOS X. Installation on Linux (Ubuntu) is supported via PyPI. Source code is available. Programming language: Python 3. Any restrictions to use by non-academics: N/A. Data availability: The dataset used in the manuscript and tutorial is available on Zenodo Dataset. Tutorial: A Jupyter notebook encompassing step-by-step Tutorial. Documentation: An online documentation explaining this package, its functions and how to use it can be found here (Documentation).
