# Supplementary material for: Protomix: a Python package for 1H-NMR metabolomics data preprocessing
Source: Bioinform Adv. 2024 Nov 27;5(1):vbae192. doi: 10.1093/bioadv/vbae192 (PMC11671038; doi:10.1093/bioadv/vbae192)
Supplement: vbae192_Supplementary_Data [file vbae192_supplementary_data.docx]

## Supplementary information file

**Protomix: A Python Package for ^1^H-NMR Metabolomics Data Pre-processing**

Mohammed Zniber^1,‡,^*, Youssef Fatihi^2^, Tan-Phat Huynh^1,^*

^1^ Laboratory of Molecular Science and Engineering, Åbo Akademi University, Turku, Finland.

^2^ Department of Computer Science, Ibn Tofail University, Kenitra, Morocco.

^‡^ Present address: Materials Informatics Laboratory, University of Turku, Vesilinnantie 5, Quantum building, Turku 20500, Finland.

*To whom correspondence should be addressed.

**Data acquisition**

Human urine aliquots of 450 µL were mixed with 50 µL of D_2_O containing 1 mM of TSP, 0.2 M of Na_2_HPO_4_ | 0.04 M of NaH_2_PO_4_ (pH 7.4) and 0.8% w/v of NaCl to a total volume of 500 µL in Eppendorf tubes. Following this, the samples underwent centrifugation at 3000 rpm for 5 minutes and the supernatants were transferred into clean 5 mm NMR tubes using fine Pasteur pipettes. Finally, the tubes were capped and labelled prior to NMR acquisition. NMR experiments were conducted on a 600 MHz NMR spectrometer (AVANCE III, Bruker, Germany) equipped with a liquid nitrogen cooled Prodigy TCI (inverted CryoProbe) at a temperature of 298 K.

**Implemented functions**

**extract_params:** This function extracts acquisition parameters from ‘acqus’ files. It serves as a vital step in the preprocessing phase, setting the stage for subsequent steps by retrieving essential data parameters.

**extract_fids:** This step is designed to extract Free Induction Decay (FID) data from a specific directory. It facilitates further preprocessing and analysis, paving the way for more advanced data manipulation and interpretation in subsequent stages of the workflow.

**group_delay_removal:** This function is responsible for correcting the group delay effects in NMR data, which are often introduced by digital filters during the data acquisition process.

**solvent_residuals_removal:** This function eliminates the influence of solvent residuals, mainly water, which can obscure signals from target molecules in the spectrum, enhancing the clarity and focus on the molecules of interest within the Protomix workflow.

**apodization:** This function enhances the spectrum's Signal-to-Noise Ratio (SNR) and/or resolution by multiplying the FID signal with a typically decaying positive signal.

**zero_filling:** This function adds zero to the end of the FID to enhance the resolution of the spectra.

**fourier_transform:** This function converts the time-domain FID signals into frequency-domain spectra in Hertz (Hz), facilitating the further conversion into chemical shifts expressed in parts per million (ppm). This transformation is a key step, aiding in the deeper analysis and interpretation of NMR data.

**internal_referencing:** This function utilizes an internal compound, such as TMSP (Tetramethylsilapropionic acid), as a reference point for calibrating the chemical shift scale in the NMR spectrum.

**phase_correction:** This function is vital in adjusting phase discrepancies in the NMR spectra, which might arise due to variations in the initial phase of the signals.

**baseline_correction:** This function is instrumental in rectifying baseline distortions in the NMR spectra, which can occur due to various instrumental and experimental factors.

**Icoshift:** This class plays a crucial role in aligning the peaks across different NMR spectra, addressing variations that might occur due to experimental or instrumental inconsistencies.

**negative_values_zeroing:** Due to imperfect phase or baseline correction, small residual negative values can still be present in the spectrum, which are not interpretable. This function identifies and set these negative values to zero to prevent potential misinterpretations and errors in subsequent data analysis.

**window_selection:** This function extracts the significant portion of the spectrum that holds valuable information, retaining it for further data analysis.

**region_removal:** This function zeroes out specific spectral regions, thereby ensuring that the analysis is concentrated on the segments of the spectrum that are of actual interest.

**binning:** This function addresses the issue of peak misalignment across spectra and helps in reducing data dimensionality. Utilizing either trapezoidal or rectangular interpolation, it creates equally-spaced bins of specified length or number.

**normalize:** This function applies different normalization methods to a DataFrame of spectra, where each row represents a sample and each column corresponds to data points in the spectrum. It supports three methods: Probabilistic Quotient Normalization (PQN), Total Area Normalization, and Standard Normal Variate (SNV).

**Figure S1:** Free induction decay of one urine sample.

**Figure S2:** Group delay (A), Removal of group delay (B), NMR spectrum before (C) and after group delay removal (D).

**Figure S3:** FID with (A) and without solvent residuals (B). NMR spectrum before (C) and after removing the solvent residuals (D).

**Figure S4:** FID after line broadening, apodization.

**Figure S5:** Resulting NMR spectrum after Fourier transform.

**Figure S6:** NMR spectrum after internal referencing.

**Figure S7:** NMR spectrum after phase correction

**Figure S8:** Removal of the water resonance region between 4.5 and 6.1.

**Figure S9:** NMR spectrum after baseline correction.

**Figure S10:** NMR spectrum regions before (A) (C) and after peak alignment (B) (D). Whole NMR spectrum after peak alignment (E).


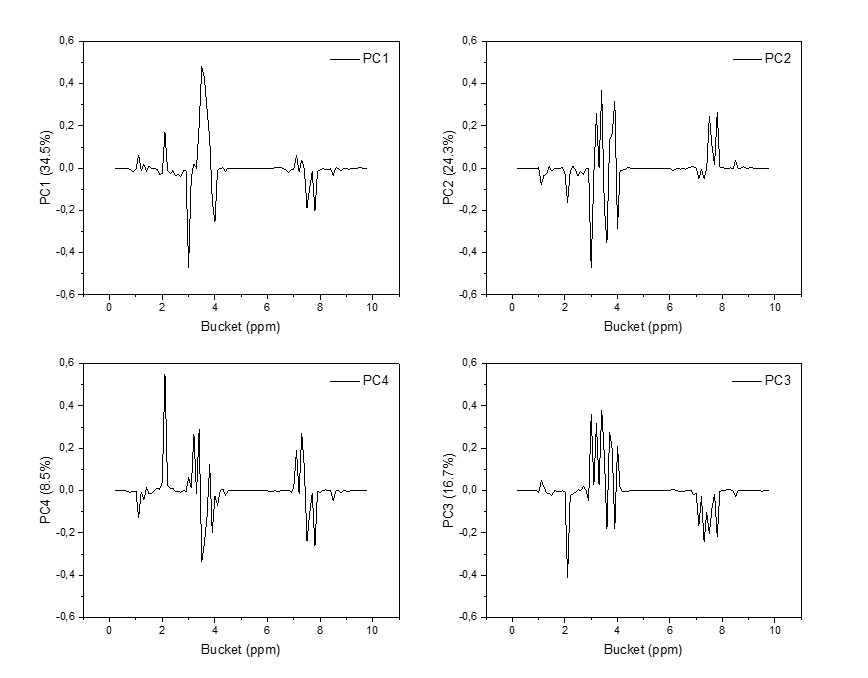


**Figure S11:** Loadings plots for the first four principal components.
